# How does livelihood capital influence the green production behaviors among professional grain farmers cultivating high-quality rice?

**DOI:** 10.3389/fnut.2025.1555488

**Published:** 2025-05-26

**Authors:** Bin Zhang, Wenyi Mao, Zhen Hu, Yingshu Cai, Ning Xie, Zhenlin Weng

**Affiliations:** ^1^School of Economics and Management, Jiangxi Agricultural University, Nanchang, Jiangxi, China; ^2^School of Humanities and Public Administration, Jiangxi Agricultural University, Nanchang, Jiangxi, China; ^3^Jiangxi Rural Revitalization Strategy Research Institute, Jiangxi Agricultural University, Nanchang, Jiangxi, China

**Keywords:** livelihood capital, professional grain farmers, green production behavior, value perception, government regulation

## Abstract

**Introduction:**

As a vital food crop in China, the sustainable production of high-quality rice is essential for ensuring food security and facilitating the green transformation of agriculture. However, the limited adoption of green production technologies for high-quality rice among farmers poses a significant obstacle to the sustainable development of the grain sector. While previous studies have primarily focused on the adoption of green technologies by traditional farmers, there has been a lack of attention on professional grain farmers as a distinct category of agricultural operators.

**Methods:**

This study, based on the sustainable livelihood framework, focuses on professional grain farmers. By analyzing data from 655 professional grain farmers in Jiangxi Province and utilizing the ordered logit model, we analyzed and validated the influence of livelihood capital on the green production behaviors. Additionally, we applied mediating and moderating effect models to clarify the roles of value cognition and government regulation in this relationship.

**Results:**

The study found the following: (1) livelihood capital significantly and positively affects the green production behaviors of professional grain farmers. Specifically, human capital, natural capital, financial capital, and social capital all contribute to the adoption of green production practices. (2) The relationships between livelihood capital and green production behavior are partially mediated by perceptions of economic, ecological, and social benefits, with the mediating effects ranked in descending order. (3) Government regulation acts as a moderator, where stronger incentive and guidance policies amplify the influence of livelihood capital on professional grain farmers’ green production behaviors. (4) Heterogeneity analysis indicates that livelihood capital and value perception have a more pronounced effect on green production behaviors among farmers with higher levels of livelihood capital.

**Discussion:**

Based on these results, the study suggests enhancing the allocation of farmers’ livelihood capital, intensifying the dissemination and education of green production technologies, and strengthening policy incentives and guidance.

## 1 Introduction

Green production is a cornerstone strategy for promoting sustainable agricultural development, ensuring food safety, and preserving rural ecological environments (1). Historically, the extensive growth model, driven by factor expansion, has contributed to short-term agricultural economic growth but has also exacerbated rural ecological degradation and agricultural non-point source pollution (2). This has not only jeopardized the living conditions of rural residents and the safety of agricultural products but has also hindered the preservation of rural environments and the transformation of the agricultural sector. In recent years, the Chinese government has increasingly prioritized the development of green agriculture, with green production serving as a guiding principle. A series of policy measures have been introduced to harmonize agricultural modernization with resource conservation, environmental sustainability, and product safety. The transformation of agricultural development models and the enhancement of green production practices have become essential approaches for the protection and management of rural ecological environments (3).

Despite the initiatives implemented by the Chinese government to enhance agricultural sustainability, agricultural sources continue to contribute to approximately 50% of the nation’s total water pollutant emissions, as indicated in the recent report from China’s second national census of pollution sources (4). Furthermore, the average application of chemical pesticides per unit area in China is 2.5 times greater than that in developed nations (5), significantly surpassing the international average (6). Meanwhile, the adoption of green production technologies among farmers remains notably low (7). Xu et al. (8) Highlighted that the distinctive characteristics of Chinese farmers resulted in fragmented, diverse, and complex agricultural production systems. Given that farmers are the primary participants in agricultural production, their sustained commitment to green practices is essential for advancing agricultural green development (9). In the context of fostering new agricultural entities, professional grain farmers, due to their specialized operations, larger production scale, and higher propensity to invest in capital, are increasingly recognized as the driving force behind grain production in China (10). In light of evolving consumer spending patterns, escalating resource and environmental constraints, and growing challenges in raising farmers’ incomes, it is imperative to strengthen the intrinsic motivation of professional grain farmers to cultivate high-quality rice and promote the development of high-quality rice production. This transition from the traditional production model of “maximizing yield through material inputs” to the green production model of “optimizing quality through technological inputs” is vital for achieving agricultural green transformation and ensuring national food security.

High-quality rice, an innovative crop variety distinguished by its high yield, superior grain quality, and robust resistance, has garnered substantial popularity among farmers. Since its introduction, it has catalyzed the transformation of rice production structures and has emerged as the predominant variety in several key rice-growing regions (11). By 2023, eight of the top 10 rice varieties with the largest planting areas nationwide were classified as high-quality rice (12). Although high-quality rice enhances yields and income for farmers, the quest for even higher yields has resulted in increased use of pesticides and fertilizers, driven by the pursuit of greater profitability (13). This trend poses risks of surface pollution and ecological degradation, thereby impeding the sustainable and environmentally friendly development of the grain sector.

Green production behavior encompass agricultural practices that not only enhance productivity and profitability but also mitigate environmental pollution in rural areas and improve resource utilization efficiency (14). Current studies have examined the factors influencing farmers’ green production behavior, revealing a consensus on the roles of farmers’ livelihood capital, individual cognition, and external contexts. Regarding livelihood capital, existing literature predominantly investigates its impact on green production behavior through various dimensions. For instance, Liu et al. (15) identified that human capital factors such as gender, education level, and age influence farmers’ green production behaviors to differing extents. Additionally, Liu et al. (15) demonstrated that social capital among rice farmers significantly facilitates the adoption of green production technologies. Ren et al. (16) utilized the double-hurdle model to show that while farmers’ natural and financial capital do not significantly affect their green production decisions, they positively influence the degree of green production behavior. Xu et al. (17) found that human, natural, economic, and social capital significantly impact farmers’ adoption of green production practices. In terms of individual cognition, Findlater (18) and Mankad (81) noted that farmers often encounter challenges in making rational decisions, primarily due to cognitive disparities among individuals. Specific cognitive factors, including ecological awareness (19), levels of green cognition (20, 21), and risk perception, play crucial roles in the implementation of green production practices (22). These studies collectively affirm the positive influence of cognitive factors in encouraging farmers to engage in green production. Concerning the external policy environment, the positive externalities associated with adopting green production technologies for environmental protection often do not align with farmers’ economic efficiency requirements, necessitating external support for widespread adoption (23). Factors influencing the adoption of green production technology primarily stem from governmental and market influences (24), including government subsidies (25), regulations (26), and market pricing (27). It is noteworthy that as farmers diversify their household income structures and adapt their livelihoods, their livelihood capital evolves, subsequently impacting their production decisions (28). Research indicates that the effect of livelihood capital on different farmers’ participation in green production varies significantly. Farmers with greater human, social, and financial capital often pursue diverse livelihood strategies, which may lead to reduced emphasis on green agriculture (29). Conversely, those with a higher proportion of physical and natural capital are more inclined to adopt green production methods for long-term benefits, as their livelihoods are closely tied to agricultural production. Furthermore, the high costs associated with green production and the potential risk of income loss deter farmers with low livelihood capital from adopting such technologies due to their limited financial capacity and risk tolerance (30). This raises the question of whether livelihood capital can effectively promote farmers’ engagement in green production behavior.

The current studies are crucial to our study, yet several areas remain underexplored. Firstly, while previous studies have examined the direct effects of various dimensions and the overall level of livelihood capital on farmers’ green production behaviors, the substitutability and complementarity among different types of livelihood capital may lead to offsetting or enhancing effects when multiple capitals interact. Thus, it is essential to explore the transmission mechanisms between these factors. Secondly, although the academic community has recognized the indirect role of ecological cognition in the relationship between livelihood capital and farmers’ green production behaviors, the influence of value cognition has been largely neglected. Thirdly, studies have examined the relationship between government regulation and farmers’ green production behaviors, the intrinsic connection between livelihood capital and government regulation has not been adequately examined. Integrating government regulation into the analysis of the relationship between livelihood capital and green production behaviors is still uncommon. Fourthly, current research on green production behaviors primarily focuses on common crops such as rice, apples, tea, and wheat, with limited attention to high-quality rice. High-quality rice, characterized by high yield, superior quality, and resistance to diseases and pests, as well as efficient nutrient absorption and drought tolerance, aligns with the goals of “less pesticide, less fertilizer, high-quality, and high-yield,” contributing to the establishment of a resource-saving and environmentally friendly agricultural system.

Building on these insights, this study, from the perspective of farmers, selected 660 professional grain farmers in Jiangxi Province, China, as the research subjects. Utilizing the Sustainable Livelihoods Framework, we employed the ordered logit model to examine the influence of professional grain farmers’ livelihood capital on their green production behaviors in high-quality rice cultivation. Additionally, we explored the interactive roles of value cognition and government regulation in this context. The research aims to provide valuable insights for promoting agricultural green transformation and fostering sustainable agricultural development.

This study presents four key contributions. First, it integrates five dimensions—human capital, natural capital, financial capital, social capital, and physical capital—to construct a multidimensional framework that comprehensively assesses the impact of livelihood capital on farmers’ behaviors. Second, the research not only investigates the direct effects of livelihood capital on green production behaviors but also examines the mediating role of value cognition and the moderating role of government regulation, thereby elucidating the underlying mechanisms through which livelihood capital influences the green production behaviors of professional grain farmers. Third, through heterogeneity analysis, the study identifies distinct behavioral patterns among professional grain farmers with varying levels of livelihood capital, which can inform the design of targeted and personalized policy interventions. Lastly, by focusing on the green production of high-quality rice, the study provides robust theoretical and empirical evidence to support the green transformation of high-quality rice production.

## 2 Theoretical framework and hypotheses

The sustainable livelihood theory originates from Sen’s concept of sustainable development, which was formulated to tackle rural poverty challenges (31). Building upon this theory, the Sustainable Livelihoods Framework, developed by the UK Department for International Development (DFID), has been widely applied (32). This framework, centered on livelihood capital, investigates how farmers formulate livelihood strategies. Recent studies have examined the relationship between livelihood capital and farmers’ willingness and actions concerning farmland transfer (33), crop planting decisions (34), and other areas, highlighting that livelihood capital significantly influences farmers’ behaviors and intentions. Specifically, as the level of livelihood capital increases and basic living needs are met, farmers tend to prioritize issues beyond their immediate livelihoods (35). Following the work of Ma et al. (36), this study categorizes the livelihood capital of professional grain farmers into human capital, social capital, natural capital, physical capital, and financial capital, incorporating these into the analytical framework for professional grain farmers’ green production behaviors. Within this framework, livelihood capital is recognized as a fundamental determinant of the livelihood strategy choices of professional grain farmers, directly affecting their behavioral capabilities (37). In the era of agricultural modernization, professional grain farmers face critical livelihood decisions concerning the transformation of agricultural development practices. Green production, as a livelihood strategy employed by these farmers to promote sustainable agricultural development, is significantly shaped by their own livelihood capital.

### 2.1 Impact of livelihood capital

Human capital comprises labor capacity, knowledge, and skills (38). Farmers with higher levels of education are more adept at recognizing the risks posed by chemical fertilizers and pesticides in the cultivation of high-quality rice, which encourages them to adopt more sustainable production practices (39). Farmers in better health are better able to increase labor input, manage the demands of cultivating high-quality rice more effectively, and invest greater effort into environmentally friendly production methods (40). Households with a larger number of agricultural laborers often have greater access to specialized knowledge and awareness of sustainable production (41), making them more likely to implement green production technologies. Thus, we propose the following hypothesis:

*H1a*: Human capital positively influences the green production behaviors of professional grain farmers cultivating high-quality rice.

Natural capital encompasses the stock of natural resources available to farmers, with land serving as the cornerstone of agricultural production and representing the most critical form of natural capital for farmers (42). Studies have demonstrated that the size of cultivated land is closely associated with farmers’ adoption of environmentally friendly production practices (40). Larger areas of cultivated land are more conducive to the implementation of advanced green agricultural technologies among grain farmers, such as precision fertilization based on soil testing and integrated water and fertilizer management (43). Generally, high-quality land can enhance grain yields, reduce dependence on chemical fertilizers and pesticides, lower costs associated with sustainable production (44), and ultimately encourage grain farmers to adopt green production technologies. Thus, we propose the following hypothesis:

*H1b*: Natural capital positively influences the green production behaviors of professional grain farmers cultivating high-quality rice.

Financial capital includes cash and easily accessible loans, among other elements (45). Professional grain farmers who adopt green production practices to cultivate high-quality rice encounter substantial risks and costs, which require enhanced financial support. Households with higher incomes are more capable of absorbing these costs and risks, making them more likely to embrace green production technologies. The availability of loans is a critical factor in determining farmers’ decisions to adopt such technologies (46). A steady provision of financial resources helps alleviate the initial costs associated with implementing green production practices for high-quality rice, thereby fostering greater willingness among farmers to adopt these practices. Thus, we propose the following hypothesis:

*H1c*: Financial capital positively influences the green production behaviors of professional grain farmers cultivating high-quality rice.

Social capital refers the social relationships and information resources accessible to farmers, including dimensions such as social trust, social participation, and social networks (47). Social trust is crucial in promoting the spread of green production technologies for high-quality rice (48). The influence of party members and village cadres, coupled with the professionalism of extension staff, enhances farmers’ confidence in adopting new technologies, thereby encouraging the implementation of green production practices. Social participation offers farmers platforms for learning and knowledge exchange, enabling them to acquire technical skills and timely guidance, which alleviates learning challenges and reduces hesitation in adopting green technologies (49). Additionally, social networks facilitate interaction and information sharing among farmers, lowering transaction costs and expediting the adoption of green production technologies (50). Thus, we propose the following hypothesis:

*H1d*: Social capital positively influences the green production behaviors of professional grain farmers cultivating high-quality rice.

Physical capital includes production equipment and living facilities for farmers, such as housing and agricultural machinery (51). Adequate physical capital enables farmers to invest in environmentally sustainable agricultural equipment and technologies, thereby enhancing labor and land productivity while optimizing resource utilization efficiency (52). The accumulation of such capital facilitates the transition toward more sustainable agricultural development models, empowering professional grain farmers to implement green production technologies for high-quality rice cultivation. Thus, we propose the following hypothesis:

*H1e*: Physical capital positively influences the green production behaviors of professional grain farmers cultivating high-quality rice.

### 2.2 Substitution and synergy effects of livelihood capital

The substitution and synergy effects among livelihood capitals can significantly influence farmers’ adoption of green production practices. The substitution effect occurs when an increase in one type of capital leads to a decrease in others (53), thereby altering the structure of livelihood capitals and potentially restricting overall income growth for farmers (54). For example, natural capital often demonstrates high substitutability with other forms of capital. Enhancing natural capital can hinder the accumulation of other capitals, resulting in an opportunity cost effect that limits farmers’ income growth potential (11). This internal substitution effect encourages farmers to prioritize long-term ecological benefits and sustainable development over short-term economic gains, thereby fostering their inclination to adopt green production technologies in high-quality rice production (36). Conversely, the synergy effect of livelihood capitals refers to the maximum complementarity and minimal or zero substitution between different types of capital (55). This effect positively facilitates the transition of farmers from agricultural to non-agricultural livelihood strategies by improving their access to non-agricultural employment opportunities and income. For instance, studies have revealed that human and financial capitals exhibit a complementary relationship among apple farmers. Higher education levels can lead to increased non-agricultural income, reducing the motivation for agricultural investment (53). Similarly, social capital has been found to significantly enhance human capital (56), increasing farmers’ employment and income sources and decreasing their reliance on grain production. Given the profit-driven nature of farmers, the pursuit of economic benefits may lead them to reduce their inclination to implement green production technologies.

In summary, the substitution effects of livelihood capitals can promote the green production behaviors of professional grain farmers, while the synergy effects may inhibit such behaviors. The overall impact of livelihood capitals on professional grain farmers thus depends on the relative strength of these two effects. If substitution effects dominate, the overall effect is positive, encouraging the green production behaviors. Conversely, if synergy effects are more pronounced, the overall effect is negative, potentially discouraging the implementation of green production practices.

### 2.3 The mediation effect of value perception

Behavioral economics posits that changes in human behavioral intentions are primarily driven by shifts in cognitive processes (57). Farmers’ perceptions of the value of green production reflect their comprehensive assessment of the benefits associated with green production technologies, which is influenced by their endowments of livelihood capital. Theoretically, as the livelihood capital of grain farmers increases, their cognitive abilities are expected to improve, enabling them to better understand the multifaceted value of green production. Consequently, grain farmers with greater livelihood capital are more likely to adopt green production technologies. Typically, differences in livelihood capital levels lead to varying cognitive perceptions among farmers, thereby affecting their behavioral decisions (58). Grain farmers with higher natural capital, who are often more dependent on grain production, are more likely to recognize the long-term benefits of green production, thereby increasing the likelihood of adopting such technologies. Higher material capital indicates sufficient agricultural funding, and wealthier farmers generally have broader perspectives, leading to a deeper appreciation of the benefits of green production practices, which in turn encourages the adoption of these technologies. An increase in livelihood capital levels will prompt shifts in farmers’ value perceptions (59). Under similar conditions, grain farmers with higher human capital are more likely to receive more systematic and comprehensive education, resulting in heightened environmental awareness and greater support for the application of green environmental technologies, thereby increasing the propensity for green production behaviors (49). Richer social capital provides access to agricultural production information through various channels, leading to higher green cognition and ultimately promoting green production behaviors (60). According to Maslow’s hierarchy of needs theory, grain farmers will only pursue higher-level needs once their basic needs are satisfied (61). Wealthy grain farmers, having met their basic material needs, will seek higher-level needs such as ecological security and food safety. Farmers with higher financial capital have weaker incentives to harm the environment and higher ecological consciousness (40), making them more inclined to implement green production practices. Thus, we propose the following hypothesis:

*H2*: Value cognition serves as a mediator in the relationship between the livelihood capital of professional grain farmers cultivating high-quality rice and their green production behaviors.

### 2.4 The moderation effect of government regulation

Government regulation involves the external constraints imposed by government agencies on professional grain farmers’ adoption of green production technologies through regulatory policies, which encompass both incentive and guidance measures (62). Incentive measures include educational and technical training, market information provision, financial support, and subsidies. These initiatives aim to enhance farmers’ educational levels, improve their cultural literacy and professional skills, alleviate financial constraints, support the expansion of production scale, and upgrade production conditions and technological investments. By increasing access to information, these incentive policies optimize farmers’ livelihood capital, reduce the marginal costs of green production, and promote the adoption of sustainable practices. On the other hand, guidance measures involve policy advocacy and training services that emphasize the importance and benefits of agricultural green production, which enhance farmers’ understanding of the economic, social, and ecological advantages of green production technologies (63), thereby increasing their willingness to adopt green production behaviors. Thus, we propose the following hypothesis:

*H3*: Government regulation serves as a moderating factor between the livelihood capital of professional grain farmers cultivating high-quality rice and their green production behaviors.

**Figure 1:**
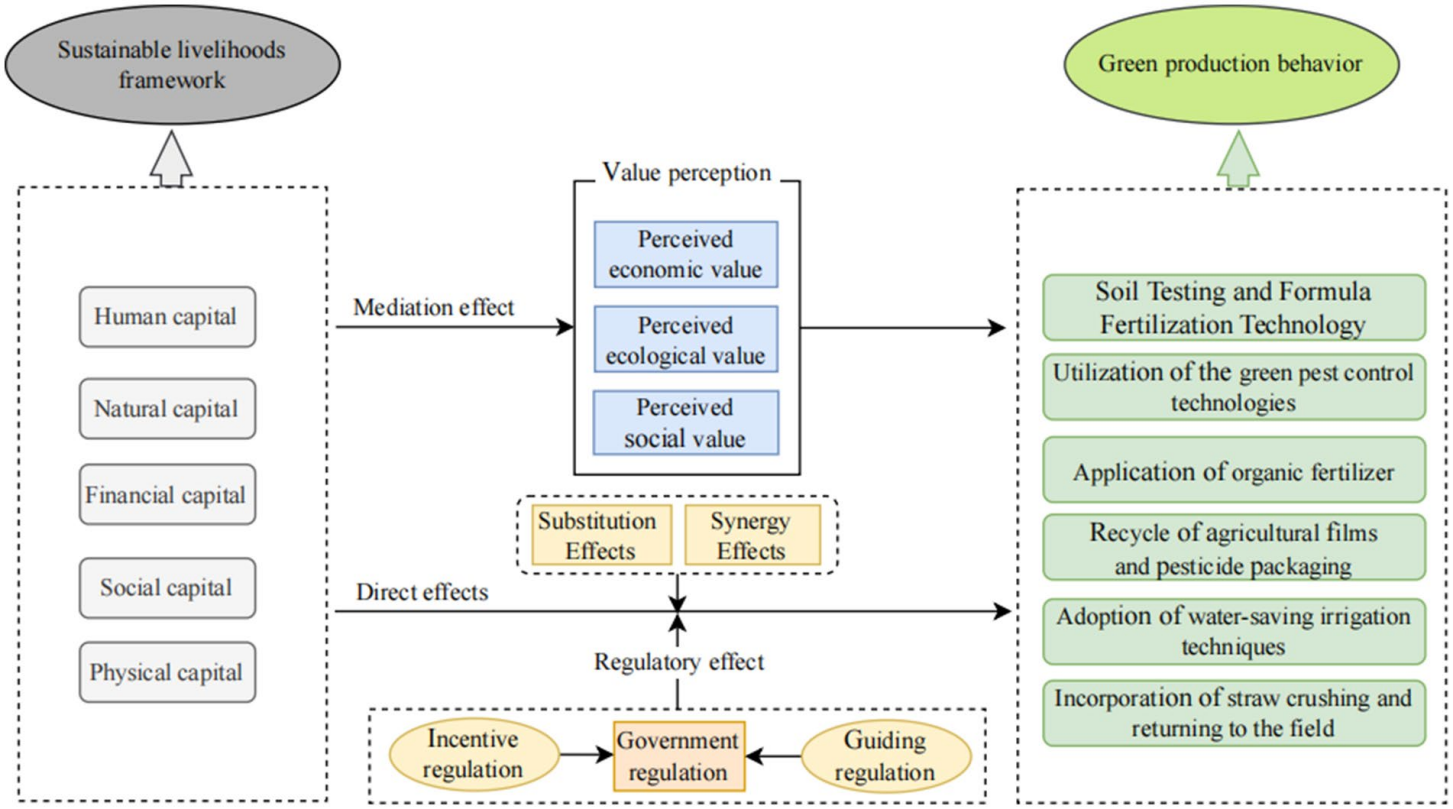
Model concept.

Based on the above analysis, we constructed a conceptual figure of the model influencing the green production behavior of high-quality rice by professional grain farmers. Figure 1 shows the conceptual figure of the model.

## 3 Data sources and model construction

### 3.1 Data sources

This study focused on Jiangxi Province in East China as the primary research area. Jiangxi was selected due to its prominence as one of China’s 13 major grain-producing regions, ranking third nationally in both rice planting area and total output. The province’s favorable natural conditions facilitate the cultivation of high-quality rice. The Jiangxi Provincial Government placed a strong emphasis on agricultural development, particularly the production of high-quality rice, as evidenced by recent policy initiatives. Consequently, Jiangxi Province serves as a representative case for analyzing the practices of professional grain farmers in cultivating high-quality rice. Given the uneven geographical distribution of professional grain farmers, the research team conducted a preliminary survey prior to the formal questionnaire investigation to refine the questionnaire design. Between June and August 2023, the team interviewed and surveyed professional grain farmers with planting areas exceeding 30 mu (approximately 2 hectares) in 77 towns in Jiangxi Province. These towns were selected from 17 major grain-producing counties, with 4–5 towns sampled from each county. A total of 691 questionnaires were distributed. After excluding those with incomplete responses, inconsistent answers, or excessively short completion times, 655 valid questionnaires were retained, resulting in a validity rate of 94.8%.

### 3.2 Variable selection and measurement

Dependent Variable: Building upon previous research and the attributes of high-quality rice varieties (64), the production activities of professional grain farmers are categorized into three stages: pre-production, in-production, and post-production. Six key indicators were selected to evaluate the green production behaviors of professional grain farmers specializing in high-quality rice, and the questionnaires were designed with the questions: “Do you implement the soil testing and precision fertilization technique?” “Do you utilize the green pest control technologies?” “Do you apply organic fertilizer?” “Do you recycle agricultural films and pesticide packaging?” and “Do you incorporate straw crushing and returning to the field?” To quantitatively assess the green production behaviors of professional grain farmers, this study employs relevant research methodologies (62), treating each of the six behaviors as a binary variable. The sum of these variables yields a comprehensive score representing each farmer’s green production behavior. The specific indicators and their levels are outlined in Table 1. Among the sample, professional grain farmers implementing 0–2 green production behaviors constitute 2.9%, those implementing 3–4 behaviors account for 23 and 44.1%, respectively, while those implementing 5–6 behaviors together constitute less than 30%. These results suggest that the level of green production behaviors among professional grain farmers in the sample is still relatively low, highlighting the urgent need to promote green production practices among professional grain farmers in high-quality rice cultivation.

**Table 1:** Construction of the indicator system for green production behavior.

	Indicator	Measurement item
Dependent variable category	Soil Testing and Formula Fertilization Technology	Yes = 1, no = 0
	Utilization of the green pest control technologies	Yes = 1, no = 0
	Application of organic fertilizer	Yes = 1, no = 0
	Recycle of agricultural films and pesticide packaging	Yes = 1, no = 0
	Adoption of water-saving irrigation techniques	Yes = 1, no = 0
	Incorporation of straw crushing and returning to the field	Yes = 1, no = 0
Dependent variable	Green production behavior	0–2 green production behaviors = 1, 3 behaviors = 2, 4 behaviors = 3, 5 behaviors = 4, 6 behaviors = 5

Core Independent Variable: These are derived from five dimensions of the livelihood capital of professional grain farmers. Human capital is assessed through education level, health status, and the proportion of the farming population. Natural capital is measured by the actual farmland area and farmland quality. Financial capital is evaluated based on household income and the ease of borrowing. Social capital is reflected through social trust, social participation, and social networks. Physical capital is measured by the number of agricultural machinery and tools owned by the farmers. Building upon prior research (37), this study utilized the entropy weight method to allocate weights to the sub-indicators that form the overall index. Specifically, all sub-indicators within the same upper-level indicator were assigned equal weights.

Mediating Variable: The mediating variable in this study is value perception. The adoption of green production practices for high-quality rice by professional grain farmers yields substantial positive externalities. These practices not only augment farmers’ income and elevate the economic value of regional brands but also mitigate agricultural non-point source pollution, thereby offering ecological benefits and enhancing the quality of agricultural products, ultimately fostering social benefits. In alignment with prior research (65), value perception is evaluated based on economic, ecological, and social benefits using a Likert five-point scale.

Moderating Variable: Government regulation serves as the moderating variable. Following the research by Guo et al. (66), government regulation is assessed through incentive regulation and guiding regulation, utilizing data from field surveys.

Instrumental Variable: The “average livelihood capital of other sample households within the same township” was utilized as an instrumental variable for the livelihood capital of professional grain farmers. This selection is based on the peer effect exerted by the average livelihood capital of other households in the township on the livelihood capital of selected professional grain farmers. Notably, this variable does not directly affect the farmers’ adoption of green production practices.

Control Variable: Based on prior research (67), the gender, age, and cooperative participation of professional grain farmers were incorporated as control variables. The definitions of these variables and their descriptive statistics are presented in Tables 2, 3.

**Table 2:** Construction of the indicator system for livelihood capital.

Dimension	Indicator	Measurement Item	Weight
Human capital	Education level	Illiterate = 1, primary school = 2, junior high school = 3, senior high school = 4, college or above = 5	0.126
	Health status	Very unhealthy = 1, relatively unhealthy = 2, general = 3, relatively healthy = 4, very healthy = 5	0.070
	Proportion of farming population	0 ~ 20% = 1, 21~40% = 2, 41~60% = 3, 61%–80% = 4, 81~100% = 5	0.108
Natural capital	Farmland area (hm^2^)	3 ~ 10 hm^2^ = 1, 11 ~ 15 hm^2^ = 2, 16 ~ 20 hm^2^ = 3, 21 ~ 25 hm^2^ = 4, above 26 hm^2^ = 5	0.045
	Farmland quality	Very poor = 1, relatively poor = 2, general = 3, relatively good = 4, very good = 5	0.022
Financial capital	Household income in 2022 (RMB)	Less than 10,000 RMB = 1, 10,001~30,000 RMB = 2, 30,001~50,000 RMB = 3, 50,001~100,000 RMB = 4, more than 100,000 RMB = 5	0.029
	Ease of borrowing	Very difficult = 1, relatively difficult = 2, general = 3, relatively easy = 4, very easy = 5	0.182
Social capital	Social trust	Degree of trust in relatives and friends: not trust = 1, slightly trust = 2, neutrally = 3, relatively trust = 4, very trust = 5	0.080
	Social participation	Frequency of participation in village organizational activities: never = 1, rarely = 2, occasionally = 3, frequently = 4, very frequently = 5	0.235
	Social network	Neighborhood relationship: very Distant = 1, relatively distant = 2, neutral = 3; relatively close = 4, very Close = 5	0.025
Physical capital	Number of agricultural machinery and tools	0 unit = 1, 1 ~ 2 units = 2, 3 units or more = 3	0.079

**Table 3:** Variable assignment and descriptive statistics.

Variable category	Variable name		Variable definition	Mean	Std.
Dependent variable	Green production behavior		Number of green production behaviors in which farmers are involved: 0–2 green production behaviors = 1, 3 behaviors = 2, 4 behaviors = 3, 5 behaviors = 4, 6 behaviors = 5	4.049	0.868
Core independent variable	Livelihood capital		Comprehensive evaluation of livelihood capital	2.603	0.505
Mediating variable	Value perception	Perceived economic value	Green production behaviors can enhance the economic benefits: Strongly disagree = 1, relatively disagree = 2, neutrally = 3, relatively agree = 4, strongly agree = 5	2.803	0.816
		Perceived ecological value	Green production behaviors contribute to the alleviation of pollution issues in production regions: Strongly disagree = 1, relatively disagree = 2, neutrally = 3, relatively agree = 4, strongly agree = 5	2.805	0.803
		Perceived social value	Green production behaviors can improve the quality and safety of rice, resulting in substantial social benefits: Strongly disagree = 1, relatively disagree = 2, neutrally = 3, relatively agree = 4, strongly agree = 5	2.913	0.853
Moderating variable	Government regulation	Incentive regulation	Government’s support in subsidizing for green production technologies: Very small = 1, small = 2, moderate = 3, large = 4, very large = 5	3.382	0.983
		Guiding regulation	Government’s support in public education and technical training for green production technologies: Very small = 1, small = 2, moderate = 3, large = 4, very large = 5	3.003	1.179
Control variable	Sex		Farmer’s sex: Female = 0, female = 1	0.370	0.483
	Age		Farmer’s actual age (years old)	3.663	1.011
	Participation in cooperatives		Whether the farmer has participated in cooperatives: No = 0, yes = 1	0.391	0.488

### 3.3 Methods

The entropy weight method is a technique used to calculate the weights of indicators by evaluating data dispersion and employing information entropy to determine the entropy weights of each indicator. This approach effectively minimizes human bias, thereby accurately reflecting the relative importance of various indicators in comprehensive assessments. This study applies the entropy weight method to evaluate the level of livelihood capital among farmers, with the calculation formula adapted from a reputable academic journal (68).

This study employed the ordered logit model to explore the factors influencing green production behaviors among professional grain farmers cultivating high-quality rice. The ordered logit model is particularly appropriate for analyzing ordered categorical data, as it can predict and elucidate the relationships between the ordered levels of the dependent variable without necessitating normality or homoscedasticity assumptions. The model is formulated as follows:


(1) $$P(y=j/x_{i})=\frac{1}{1+e^{-(\alpha_{j}+\beta_{\mathrm{xi}})}}$$

In Equation (1), *y* denotes the degree of implementation of green production practices for high-quality rice. *j* denotes the level of implementation of these practices by professional grain farmers in the cultivation of high-quality rice, where *j* ranges from 1 to 5, corresponding to the categories of “very satisfied,” “satisfied,” “neutral,” “dissatisfied,” and “very dissatisfied,” respectively. The factors influencing the adoption of green production practices for high-quality rice by professional grain farmers are denoted by *x_i_*. The cumulative model is formulated as follows:


(2) $$\log\mathrm{it}(p_{j})=\ln[\frac{p(y\leq j)}{p(y\geq j+1)}]=\alpha_{j}+\mathrm{\beta X}+\varepsilon$$

In Equation (2), *p*_*j* =_ *p* (*y* = *j*), *j* = 1, 2, 3, 4, 5. *β* denotes the regression coefficient associated with each independent variable, while *α_j_* represents the intercept term. Using the estimated values of *α_j_* and *β*, the probability formula for a particular scenario can be derived from Equation 3:


(3) $$P(y=\frac{j}{x_{i}})=\frac{e^{-(\alpha_{j}+\beta_{\mathrm{xi}})}}{1+e^{-(\alpha_{j}+\beta_{\mathrm{xi}})}}$$

Investigating the mediating role of value perception provides a comprehensive understanding of how livelihood capital influences the green production behaviors of high-quality rice among professional grain farmers. The mediating effect operates when the independent variable (*X*) not only directly affects the dependent variable (*Y*) but also indirectly impacts *Y* through its impact on the mediating variable (*M*). In this context, *M* serves as the mediating variable. Therefore, the following mediating effect model is proposed:


(4) $${\mathrm{GT}}_{i}=\alpha_{0}+\alpha_{1}X_{i}+\sum \alpha_{2}Z_{i}+\varepsilon_{1}$$

(5) $${\mathrm{EE}}_{i}=\beta_{0}+\beta_{1}X_{i}+\sum \beta_{2}Z_{i}+\varepsilon_{2}$$

(6) $${\mathrm{GT}}_{i}=\gamma_{0}+\gamma_{1}X_{i}+\gamma_{2}{\mathrm{EE}}_{i}\sum \gamma_{3}Z_{i}+\varepsilon_{3}$$

In Equations (4–6), *GT_i_* represents the green production behaviors of high-quality rice among professional grain farmers, *EE_i_* denotes the value perception, *X_i_* signifies the livelihood capital, and *Z_i_* is the control variable. The parameters to be estimated are denoted by *α*, *β*, and *γ*, with *ε*_1_, *ε*_2_, and *ε*_3_ representing the error terms. Equation (4) describes the impact of livelihood capital on the green production behaviors; Equation (5) explains the effect of livelihood capital on value perception; Equation (6) investigates the influence of value perception on the green production behaviors.

To examine how the government regulation moderates the relationship between livelihood capital and grain farmers’ green production behaviors, the following moderation effect model is proposed:


(7) $${\text{Wish}}_{i}=\delta_{0}+\delta_{1}{\mathrm{Lc}}_{i}+\delta_{2}{\mathrm{Trs}}_{i}+\delta_{3}{\mathrm{Lc}}_{i}\times {\mathrm{Trs}}_{i}+\beta_{4}{\text{Control}}_{i}+e_{4}$$

In Equation (7), *Trs_i_* denotes the government regulation applied to the *i*-th farmer. The moderating effect is assessed by examining the coefficient of the interaction term between livelihood capital and government regulation (*Lc_i_* × *Trs_i_*).

## 4 Empirical evidence and analysis of results

### 4.1 Factors influencing the green production behaviors of high-quality rice among professional grain farmers

Prior to model estimation, we conducted preliminary tests on the data, following the research design established by Luo et al. (69). The findings indicated that the Cronbach’s α value for the scale was 0.7488, demonstrating high reliability. The mean variance inflation factor (VIF) was 1.47, with a maximum value of 2.09, indicating no issues with multicollinearity among the independent variables in the model. Additionally, the *p*-value from White’s test was 0.4158, suggesting that the model did not exhibit heteroskedasticity. Confirmatory factor analysis (CFA) revealed a good fit for the model, with significant estimates for the mean and variance of all variables. Furthermore, Harman’s single-factor test suggested a low probability of common method bias. These results confirm that the data utilized in this study passed the initial tests and is appropriate for further analysis. To investigate the impact of livelihood capital on the green production behavior of vocational food farmers producing quality rice, we employed an ordered Logit model for estimation, with the results presented in Table 4.

**Table 4:** Regression analysis of factors influencing high-quality rice planting practices among professional grain farmers.

Variable	Model 1	Model 2	Model 3	Model 4	Model 5	Model 6
Livelihood capital	0.467^**^ (0.145)	0.459^**^ (0.144)				
Human capital			0.305^**^ (0.111)	0.301^**^ (0.111)		
Education level					0.424^***^ (0.119)	0.396^**^ (0.118)
Health status					0.226^**^ (0.086)	0.228^**^ (0.086)
Proportion of farming population					0.160^**^ (0.076)	0.162^**^ (0.076)
Natural capital			0.318^***^ (0.090)	0.312^**^ (0.090)		
Farmland area					0.269^**^ (0.084)	0.265^**^ (0.084)
Farmland quality					0.315^**^ (0.118)	0.305^**^ (0.117)
Financial capital			0.654^***^ (0.167)	0.649^***^ (0.165)		
Household income in 2022					0.301^**^ (0.121)	0.295^**^ (0.120)
Ease of borrowing					0.305^**^ (0.121)	0.302^**^ (0.121)
Social capital			0.324^***^ (0.091)	0.340^***^ (0.091)		
Social trust					0.308^***^(0.086)	0.310^***^(0.086)
Social participation					0.236^**^(0.103)	0.232^**^(0.102)
Social network					0.334^**^(0.101)	0.349^**^(0.101)
Physical capital			−0.032 (0.160)	−0.044 (0.159)		
Number of agricultural machinery and tools					0.118 (0.186)	0.085 (0.185)
Sex	−0.238 (0.150)		−0.182 (0.152)		−0.140 (0.153)	
Age	0.121^*^ (0.073)		0.122^*^ (0.074)		0.135^*^(0.074)	
Participation in cooperatives	0.146 (0.149)		0.138 (0.152)		0.195 (0.153)	
Prob > chi2	0.003	0.001	0.000	0.000	0.000	0.000
Pseudo R^2^	0.010		0.034		0.039	

*, **, and *** indicate the significate level of 10%, 5%, and 1%, respectively.

From Model 1, the coefficient of livelihood capital is positive and significant at the 5% level, indicating a robust positive relationship between the livelihood capital of professional grain farmers and their adoption of green production practices for high-quality rice. One possible explanation is that, due to the limited stock of livelihood capital among professional grain farmers in the study region (with an average value of only 2.603), the substitution effect within livelihood capital outweighs the synergistic effect, thereby restricting income growth. This situation compels farmers to focus more on the production and management of high-quality rice. By adopting green production technologies, farmers can achieve sustainable production of high-quality rice and secure long-term economic benefits. Model 3 shows that natural capital, financial capital, and social capital have significant effects at the 1% level, while human capital has a significant effect at the 5% level, with all impact coefficients being positive. This suggests that an increase in these four types of capital significantly enhances the green production behavior of professional grain farmers specializing in high-quality rice. Consequently, hypotheses H1a, H1b, H1c, and H1d are supported. In contrast, physical capital does not have a significant impact on the green production behaviors of professional grain farmers cultivating high-quality rice, thus failing to validate hypothesis H1e.

Regarding control variables, the gender of professional grain farmers did not exhibit a significant effect, potentially due to the characteristics of the sample data. In this study, male professional grain farmers comprised 61.9% of the total sample, indicating an overrepresentation. Although participation in cooperatives was found to positively influence the green production of high-quality rice, this effect did not reach statistical significance. One possible reason for this lack of significance could be that cooperatives fail to provide sufficient training in production technologies for the participating professional food farmers. As a result, this deficiency may impede farmers’ comprehension of green rice production and not significantly affect their production decision-making, which explains the observed insignificance of this variable. Age significantly influences the adoption of green production practices in high-quality rice cultivation at the 10% level. This trend can be attributed to the prevalent rural practice where younger individuals often work outside, leaving middle-aged and older individuals to manage farming activities. The sample data show that professional grain farmers under 40 years old constitute only 11.7% of the total. Additionally, as professional grain farmers grow older, they accumulate more farming experience and knowledge, facilitating a deeper understanding of the benefits associated with green production technologies. Consequently, they are more inclined to adopt such practices to enhance farming efficiency.

### 4.2 Mediation effect test of value perception

As presented in Table 5, the value perception has successfully passed both the Sobel and Bootstrap tests, confirming its significant mediating role between livelihood capital and green production behaviors among professional grain farmers cultivating high-quality rice. Thus, Hypothesis H2 is verified. However, the mediating effects of value perception differ across various dimensions. Specifically, the mediating effect of perceived economic value is significant at the 5% level, accounting for 22.6% of the total effect. This suggests that an increase in livelihood capital enhances farmers’ perception of economic benefits, thereby encouraging them to adopt green production practices for high-quality rice. Similarly, the mediating effect of perceived ecological value is significant at the 10% level, indicating the existence of a pathway: “livelihood capital → ecological benefit cognition → green production behavior of high-quality rice,” with a mediating effect proportion of 21.7%. Additionally, perceived social value exhibits significance at the 5% level, demonstrating that farmers’ social benefit cognition plays a significant partial mediating role in the relationship between livelihood capital and their green production behaviors for high-quality rice.

**Table 5:** Mediation effect test results of value perception.

Variable	Step 1	Step 2	Step 3	Sobel test (Z-value)	Bootstrap verification (confidence Interval)	Mediation effect proportion%
Livelihood capital	0.204^**^ (0.067)	0.478^***^ (0.060)	0.155^**^ (0.070)	2.134^**^	[0.002, 0.057]	22.6
Perceived economic value			0.101^**^ (0.044)			
Livelihood capital	0.204^**^ (0.067)	0.456^***^ (0.061)	0.159^**^ (0.070)	2.171^*^	[0.005, 0.055]	21.7
Perceived ecological value			0.099^**^ (0.043)			
Livelihood capital	0.204^**^ (0.067)	0.398^***^ (0.065)	0.155^**^ (0.069)	2.119^**^	[0.001, 0.042]	14.9
Perceived social value			0.122^**^ (0.041)			

*, **, and *** indicate the significate level of 10%, 5%, and 1%, respectively.

### 4.3 Moderation effect test of government regulation

This study integrated an interaction term between the livelihood capital of professional grain farmers and government regulations into the benchmark regression model for analysis, with the results presented in Table 6. The coefficient of the interaction term between professional grain farmers’ livelihood capital and incentive regulation is 0.406, statistically significant at the 1% level. This outcome indicates that government incentive regulations exert a significant positive moderating effect on the relationship between livelihood capital and the green production behaviors of professional grain farmers. Specifically, an increase in the intensity of incentive regulations enhances the positive impact of livelihood capital on the green production behaviors. Additionally, the coefficient of the interaction term between professional grain farmers’ livelihood capital and guiding regulations is 0.339, which is also statistically significant at the 1% level. This suggests that guiding regulations positively moderate the relationship, implying that stronger guidance regulations amplify the influence of livelihood capital on the green production behaviors. Thus, Hypothesis H3 is validated.

**Table 6:** Moderation effect test results of government regulation.

Variable	Model 7	Model 8
Livelihood capital	0.941^***^ (0.145)	1.053^***^ (0.111)
Incentive regulation	0.166 (0.111)	
Guiding regulation		0.517^***^ (0.095)
Livelihood capital × Incentive regulation	0.406^***^ (0.042)	
Livelihood capital × Guiding regulation		0.339^***^ (0.034)
Control variables	Yes	Yes
Pseudo R^2^	0.087	0.088

*, **, and *** indicate the significate level of 10%, 5%, and 1%, respectively.

### 4.4 Heterogeneity analysis

The diversity in the livelihood capital of farmers plays a crucial role in influencing their selection of livelihood strategies. To elucidate this influence, this study utilizes SPSS 22.0 software to standardize five key indicators of capital: human, natural, financial, physical, and social, specifically within the realm of vocational food and agriculture. Subsequently, K-means clustering analysis is employed to divide the sample of professional grain farmers into two distinct categories: one group consisting of 236 households with high livelihood capital and another group comprising 419 households with low livelihood capital. To determine whether there are significant differences between these two groups across the five indicators, the Mann–Whitney test, a nonparametric statistical method, is further conducted. The outcomes of this analysis are presented in Table 7.

**Table 7:** Heterogeneity analysis results.

Variable	Green production behavior									
	High livelihood capital group					Low livelihood capital group				
Livelihood capital	0.183^*^ (0.115)	0.184^*^ (0.113)	0.210^*^ (0.114)	0.934^**^ (0.273)	1.388^***^ (0.214)	0.108 (0.093)	0.119 (0.092)	0.093 (0.089)	0.933^***^ (0.174)	0.866^***^ (0.131)
Perceived economic value	0.160^**^ (0.070)					0.066 (0.060)				
Perceived ecological value		0.178^*^ (0.067)					0.045 (0.058)			
Perceived social value			0.125^*^ (0.068)					0.117^**^ (0.051)		
Livelihood capital × Incentive regulation				0.387^***^ (0.076)					0.416^***^ (0.051)	
Livelihood capital × Guiding regulation					0.233^***^ (0.063)					0.403^***^ (0.041)
Control variables	Yes	Yes	Yes	Yes	Yes	Yes	Yes	Yes	Yes	Yes
Pseudo R	0.067	0.074	0.060	0.087	0.086	0.014	0.013	0.024	0.087	0.090
Observations	236	236	236	236	236	419	419	419	419	419

*, **, and *** indicate the significate level of 10%, 5%, and 1%, respectively.

## 5 Discussion

### 5.1 Endogeneity discussion

This study examined the impact of livelihood capital among professional grain farmers on their adoption of green production practices for high-quality rice. A critical consideration is the potential for reverse causality between livelihood capital and green production behavior. Specifically, an increase in livelihood capital may encourage farmers to adopt green production methods, which forms the central hypothesis of this research. Conversely, the adoption of green production practices by grain farmers may also reshape the structure and scale of their livelihood capital, as suggested by the DFID Sustainable Livelihoods Framework, indicating the presence of reverse causality. Furthermore, unobserved omitted variables and measurement errors could influence the decision-making processes of professional grain farmers, potentially leading to biased estimation results. To address these challenges and obtain consistent estimates, this study drew on relevant literature and utilized the “average livelihood capital of other sample farmers within the same township” as an instrumental variable for the livelihood capital of professional grain farmers (70). This approach is supported by two key reasons: first, grain farmers’ production decisions exhibit a peer effect, and since farmers within the same township share similar living and cultivation conditions, their livelihood capital structures and scales are highly consistent, satisfying the relevance condition; second, the livelihood capital of other farmers does not directly affect the green production behavior of a specific professional grain farmer, meeting the exogeneity condition.

Table 8 presents the results of the instrumental variable regression analysis. The weak instrument test indicates that the Cragg-Donald Wald F-statistic is 67.473, significantly exceeding the 10% critical value of 16.380 from the Stock-Yogo test. This outcome passes the weak instrument test. Additionally, the K-P LM statistic rejects the null hypothesis at the 1% significance level, confirming the validity of the instrumental variables. After accounting for endogeneity, the regression coefficient of livelihood capital for professional grain farmers on their green production behavior of high-quality rice becomes significant at the 10% level, demonstrating the robustness of the baseline model results.

**Table 8:** Instrumental variable regression analysis results.

Variable	Stage 1	Stage 2
Livelihood capital		0.39^*^(0.221)
Average livelihood capital of other sample farmers within the same township	0.21^***^(0.024)	
Control variables	Yes	Yes
Anderson canon. Corr. LM statistic	67.473^***^	
Weak IV test	74.647 (16.380)	
Observations	655	655
Pseudo R^2^		0.098

*, **, and *** indicate the significate level of 10%, 5%, and 1%, respectively.

### 5.2 Robustness test

To evaluate the reliability of the model’s outcomes, this study conducted robustness tests by substituting the original model with the Oprobit and OLS models. The underlying rationale is that if the core explanatory variables retain their significance and the signs of the coefficients remain consistent across different model specifications, it suggests that the regression results of the original model are robust (65). As shown in Model 9-Model 16 (see Table A1), the signs of the coefficients for the core explanatory variables are entirely consistent, and there is no notable variation in their levels of significance, thereby confirming the robustness of the results.

### 5.3 Consistency with existing research

This study investigated the farmer’s adoption of green production behaviors. The analysis reveals that these behaviors are primarily concentrated in three categories and four specific practices, suggesting that their engagement remains suboptimal and requires further enhancement. This finding aligns with the research of Du et al. (71) and Benitez-Altuna et al. (72). The study also confirmed that the substitution effect among various types of livelihood capital would encourage professional grain farmers to implement green production practices, consistent with the research of Zhou et al. (73). The substitution effect within livelihood capitals was found to be stronger than the synergistic effect, which limits farmers’ income growth and increases their dependence on land for social security. This, in turn, enhances their willingness to adjust land use to boost household income.

Additionally, the study revealed that different types of livelihood capitals have varying impacts on the green production behaviors of professional grain farmers. Firstly, human capital was found to positively influence the adoption of green production behaviors, aligning with the findings of Ren et al. (16, 68). Professional grain farmers engaged in large-scale operations of high-quality rice rely heavily on the quantity and quality of labor. Farmers with higher levels of human capital demonstrate stronger awareness of ecological environmental protection and are more proficient in adopting and implementing new technologies. As a result, they are more inclined to adopt low-pollution, high-efficiency green production technologies, thereby fostering sustainable agricultural practices. However, some studies have indicated that human capital may negatively affect farmers’ energy-saving investment behaviors (74). Secondly, natural capital was also found to significantly influence the green production behavior of professional grain farmers positively, consistent with the research of Xu et al. (75). A high level of natural capital reflects favorable agricultural production conditions. This enables professional grain farmers to reduce production costs, enhance economic benefits, and strengthen long-term expectations, thereby encouraging them to adopt sustainable agricultural methods. Similarly, financial capital was observed to promote the implementation of green production practices among professional grain farmers. Farmers with higher financial capital possess strong economic strength and risk-bearing capacity (76), allowing them to absorb the risks associated with green production. Consequently, they are more inclined to adopt green production behaviors. Lastly, social capital was found to positively influence the adoption of green production practices by professional grain farmers. Social capital expands the channels through which farmers access information, facilitating the acquisition of knowledge about green production technologies and the benefits of high-quality rice production (47). Therefore, greater social capital enhances the likelihood of implementing green production behaviors, consistent with the findings of Liu et al. (15). In contrast, this study revealed that physical capital does not significantly impact the green production behavior of grain farmers, which differs from other studies highlighting the importance of physical capital in farmers’ livelihood strategy choices (77). This discrepancy may stem from the fact that professional grain farmers in the study area predominantly use conventional small agricultural machinery (74.8% possess 1–2 units, while only 15.3% have 3 or more). Such machinery is limited to basic production processes and cannot fully support the comprehensive production cycle required for high-standard green production of high-quality rice.

The mediation effect analysis demonstrated that value perception mediates the relationship between the livelihood capital of professional grain farmers and their adoption of green production behaviors. Livelihood capital enhances farmers’ value perception of green production, thereby indirectly promoting the adoption of such practices, which aligns with the findings of Sun et al. (58). The mediation effects were found to be strongest for perceived economic value, followed by perceived ecological value and perceived social value. This suggests that the perception of economic benefits plays the most critical role in how livelihood capital influences farmers’ decisions to adopt green production practices. This may be due to the fact that abundant livelihood capital provides farmers with the resources and capabilities to implement green production technologies. Consequently, they are better able to access information and technical support for green production, mitigate risks, and strengthen their perception of the economic benefits associated with these technologies. This finding is consistent with the research of Guo et al. (78), which emphasizes that the perceived economic value is a central determinant of farmers’ production decisions, directly affecting economic returns and production costs, and thus significantly influencing their green production behaviors.

The moderation effect analysis demonstrated that both incentive regulation and guiding regulation amplify the positive impact of livelihood capital on green production behaviors, consistent with the findings of Yang (26) and Xian (79). Incentive regulations, including government-provided insurance or subsidies, effectively guide farmers in optimizing the allocation of their livelihood capital. These incentives mitigate potential economic losses associated with transitioning to green production, thereby reducing risks and encouraging greater participation in sustainable agricultural practices. In contrast, guiding regulations, which involve technical training and knowledge dissemination, enhance farmers’ production capabilities and environmental awareness. This improved understanding of the benefits of green production facilitates the adoption of sustainable techniques more readily.

Heterogeneity analysis indicated that for farmers with high livelihood capital, both their livelihood capital and value perception significantly positively influence the adoption of green production behaviors. Furthermore, the mediation effect of “livelihood capital → value perception → green production behavior” is evident, with government regulation positively moderating this relationship. Conversely, for farmers with low livelihood capital, the mediation effect of value perception between livelihood capital and green production behavior is absent. This is consistent with the study by Li et al. (64). This discrepancy can be attributed to the limited resources available to farmers in the low livelihood capital group, who lack the necessary financial and technical support for green production (40). Additionally, due to insufficient information and education, farmers in the low livelihood capital group may have a less comprehensive understanding of the benefits of green production practices, which affects their willingness to adopt such practices. Despite the limitations in livelihood capital and value perception among farmers in the low livelihood capital group, government regulation continues to play a positive moderation role, as the essential support and incentives provided by the government can help these farmers overcome resource constraints, thereby facilitating the adoption of green production behaviors (75).

### 5.4 Limitations of the study and future work

Although this study enriches existing research to a certain extent, it has certain limitations. Firstly, the findings are derived from data collected from a small-scale region, specifically Jiangxi Province, China. There are notable disparities in the levels of livelihood capital and the adoption of green production practices among farmers across different countries and regions, which may restrict the applicability of our results to other contexts. To improve the generalizability and scientific rigor of our findings, it is crucial to expand the survey area and increase the sample size in future research. Furthermore, our assessment of the social networks of professional grain farmers was based on neighborhood relationships, which may not fully encapsulate this variable. Future research could consider evaluating the number of friends and relatives with stable connections instead. Secondly, while we highlight the positive moderating role of incentive and guidance regulations in government oversight on the relationship between livelihood capital and farmers’ green production behaviors, we have not examined the influence of government constraint regulations. Previous studies indicate that such constraints also affect farmers’ behaviors (80). Future research should incorporate constraint regulations into the analytical framework to provide a more comprehensive understanding of the moderating effects of government regulation in this domain. Lastly, this study aimed to explore the mediating effects of various dimensions of value perceptions between farmers’ livelihood capital and green production behaviors. We utilized the traditional three-step approach to construct the mediation model. Although we subsequently employed the Sobel and Bootstrap methods to validate the mediation results, there remains a potential for model estimation bias. Therefore, future research could benefit from utilizing alternative methods, such as the two-step approach or the KHB method, to more robustly assess mediation effects.

## 6 Research findings and policy implications

Based on the survey data from 655 professional grain farmers in Jiangxi Province, this study investigated the influence of livelihood capital on the green production behaviors of high-quality rice among grain farmers. The findings reveal the following: (1) Livelihood capital significantly promotes the green production behavior of high-quality rice among professional grain farmers. However, the effects of different types of livelihood capital differ. Human capital, natural capital, financial capital, and social capital are positively correlated with green production behavior, while physical capital does not exhibit a significant effect. (2) Value perception plays a significant mediation role between livelihood capital and green production behavior. Specifically, perceived economic value, perceived ecological value, and peiveived social value all partially mediate this relationship, with their effects ranked in descending order. (3) Government regulation moderates the direct relationship between livelihood capital and green production behaviors. Both incentive regulation and guiding regulation increase the likelihood of adopting green production practices. (4) Heterogeneity analysis indicates that for farmers with high livelihood capital, both their livelihood capital and value perception significantly positively influence the adoption of green production behaviors. In contrast, for farmers with low livelihood capital, the mediation effect of value perception between livelihood capital and green production behavior is absent.

To effectively promote the adoption of green production practices among professional grain farmers and ensure sustainable livelihoods, the following recommendations are proposed: (1) Enhance the accumulation of livelihood capital among farmers and optimize its structural configuration. Firstly, the government should actively implement vocational training programs in both agricultural and non-agricultural sectors, strengthen policy support for microenterprises, and enhance their capacity to absorb surplus rural labor, thereby diversifying income sources for farming households. Secondly, an institutional incentive mechanism should be established to improve the quality and pricing system of green agricultural products, leveraging market price signals to encourage farmers to adopt green production practices, which will effectively enhance their human and financial capital. Additionally, the government should expand subsidies for agricultural machinery and enforce land protection policies to preserve the quality and quantity of arable land while improving soil fertility, thus increasing farmers’ material and natural capital. Furthermore, the promotion of local agricultural cooperatives should be prioritized, encouraging more farmers to participate in these cooperatives to strengthen their social capital. Meanwhile, village committees should actively involve farmers in collective village activities, facilitating information exchange and communication among them to build mutual trust and further enhance their social capital. (2) Strengthen educational programs aimed at promoting sustainable production technologies. It is crucial to foster collaboration between research institutions, universities, and farmers. Providing technical training can enhance farmers’ understanding of the advantages of sustainable production, ultimately increasing their trust and acceptance of green production technologies. (3) Optimize policy design to accommodate the differences in farmers’ livelihood capital. For instance, for professional grain farmers in the high livelihood capital, efforts should focus on enhancing their understanding of the advantages of green production. This can be accomplished through technical training, the establishment of demonstration sites, and the optimization of the policy support system to promote the adoption of green technologies. In contrast, for professional grain farmers in the low livelihood capital, the priority should be on strengthening government regulations and policy guidance. Enhancing incentives for participation in green production can be achieved through ecological compensation, subsidies, and other supportive measures.

## Data Availability

The raw data supporting the conclusions of this article will be made available by the authors, without undue reservation.

## Appendix

**Table A1:** Robustness test result.

	Oprobit				Tobit			
Variable	Model 9	Model 10	Model 11	Model 12	Model 13	Model 14	Model 15	Model 16
Livelihood capital	0.250^* *^(0.083)	0.251^**^ (0.083)			0.204^**^(0.067)	0.206^**^(0.067)		
Human capital			0.167^**^ (0.063)	0.166^**^ (0.063)			0.131^**^ (0.049)	0.130^**^ (0.049)
Natural capital			0.165^**^ (0.052)	0.162^**^ (0.052)			0.131^**^ (0.041)	0.128^**^ (0.041)
Financial capital			0.350^***^ (0.095)	0.348^***^ (0.095)			0.276^***^ (0.074)	0.276^***^ (0.074)
Social capital			0.177^**^ (0.051)	0.185^***^ (0.051)			0.139^**^ (0.040)	0.146^***^ (0.040)
Physical capital			−0.006 (0.092)	−0.014 (0.091)			−0.006 (0.072)	−0.012 (0.072)
Sex	−0.101 (0.087)		−0.066 (0.087)		−0.086 (0.070)		−0.055 (0.068)	
Age	0.062 (0.042)		0.058 (0.042)		0.051 (0.034)		0.046 (0.033)	
Participation in cooperatives	0.072 (0.085)		0.081 (0.086)		0.059 (0.069)		0.064 (0.067)	
Prob > chi2	0.0105	0.002	0.000	0.000	0.009	0.002	0.000	0.000
Pseudo R^2^	0.008		0.030		0.008		0.031	0.029

*, **, and *** indicate the significate level of 10%, 5%, and 1%, respectively.
